# Novel Use of RegJointTM in the Management Talocalcaneal Coalition Interpositional Arthroplasty

**DOI:** 10.7759/cureus.97405

**Published:** 2025-11-21

**Authors:** Canan Metin, Shah Khan, Henry Atkinson

**Affiliations:** 1 Trauma and Orthopaedic Surgery, North Middlesex University Hospital, London, GBR; 2 Trauma and Orthopaedics, North Middlesex University Hospital, London, GBR

**Keywords:** adolescent athlete, foot and ankle surgery, interposition arthroplasty, regjoint implant, synthetic implant, talocalcaneal coalition, tarsal coalition

## Abstract

RegJoint™ is a bioresorbable porous polylactide scaffold that has previously been used as an interposition device in small joint arthritis of the hand and great toe. The preformed design of RegJoint™ offers the technical advantages over autologous grafts by maintaining a consistent joint space volume during healing. This case represents the first documented use of a RegJoint™ implant as an interposition arthroplasty in the treatment of symptomatic talocalcaneal coalition. There were no complications, and the patient was able to return to pain-free light exercise after three months, with a return to full contact sports after six months. The device offers potential advantages, such as precise sizing and a reduction in donor-site morbidity. The mid-term outcome in our patient suggests that this technique might provide, at the very least, comparable pain relief and functional recovery to established methods.

## Introduction

Tarsal coalition is a congenital union between two or more tarsal bones and is estimated to occur in 1-2% of the population [1]. The most common subtypes are talocalcaneal (TC) and calcaneonavicular (CN) coalitions, which together account for approximately 90% of all cases [2]. Patients typically present during adolescence with activity-related pain, recurrent ankle sprains, or restricted subtalar motion.

Initial management is conservative, focusing on activity modification, orthoses, and physiotherapy. In cases where symptoms persist despite non-operative measures, surgical intervention is considered. The main surgical approaches are coalition resection or arthrodesis, with the choice depending on the extent of joint involvement and degeneration. Resection, with or without interposition, is generally indicated in patients who have preserved joint architecture, minimal deformity, and less than 50% posterior facet involvement [3].

Following resection, an interpositional material may be used to maintain the resected gap and reduce the risk of reossification. A variety of materials have been described - including autologous fat, muscle, tendon, and bone wax [4,5] - though evidence remains inconclusive as to whether interposition offers superior outcomes compared to resection alone, or which material best minimises recurrence [6]. Traditional autologous options, while readily available, can be associated with donor-site morbidity, variable resorption, and inconsistent long-term results.

Synthetic bioresorbable implants such as RegJoint™ have been developed to address these limitations. RegJoint™ (Scaffdex Oy, Tampere, Finland) is a porous, disk-shaped, bioabsorbable interpositional implant composed of poly-L/D-lactic acid (PLDLA; 96% L/4% D) [7]. It is designed to maintain joint space following resection and to facilitate fibrous tissue ingrowth, forming a functional pseudoarthrosis-like structure [8]. The material's porous structure allows fibroblasts to enter and form dense fibrous tissue. This new tissue provides temporary support without restricting joint movement. Structural integrity is maintained for several months postoperatively, followed by gradual resorption and complete degradation within two to three years [9]. As the implant is replaced by host tissue, a biologically integrated, motion-preserving reconstruction is achieved without leaving permanent foreign material [10].

The theoretical advantages of RegJoint™ lie in its biocompatibility, controlled degradability, and ability to promote stable fibrous interposition. Its bioresorbable nature avoids long-term complications associated with metallic or non-degradable implants, such as migration or chronic foreign body reaction [9]. Maintaining joint space during healing prevents osseous re-bridging and allows for progressive tissue remodelling, resulting in a flexible, pain-free interface that preserves motion and reduces postoperative stiffness [11].

In the setting of TC coalition resection, maintaining subtalar joint space and preventing bony re-bridging are key challenges. RegJoint™ implant may offer several advantages in this context. It is temporary, but stable interposition can preserve separation between the talus and calcaneus during healing, while its capacity to facilitate fibrous tissue ingrowth could reduce the risk of osseous recurrence [11].

Notably, no published studies have utilised a RegJoint™ implant for interposition arthroplasty. Here, we present the first documented case of successful treatment using RegJoint™ in a 14-year-old male.

## Case presentation

A 14-year-old male patient presented to a specialist foot and ankle clinic with a three-year history of gradually worsening medial hindfoot pain. His symptoms were particularly aggravated by sporting activities and prolonged weight bearing, with only partial relief achieved through rest. Despite an extended period of conservative management, he experienced only temporary improvement before symptoms recurred.

On examination, the patient demonstrated localised tenderness over the sustentaculum tali with restricted subtalar joint motion compared to the contralateral limb. Neurovascular examination was unremarkable. Notably, there was a mild but correctable pes planus deformity. His subtalar joint motion was stiffer and significantly reduced compared with the contralateral side.

**Figure 1:**
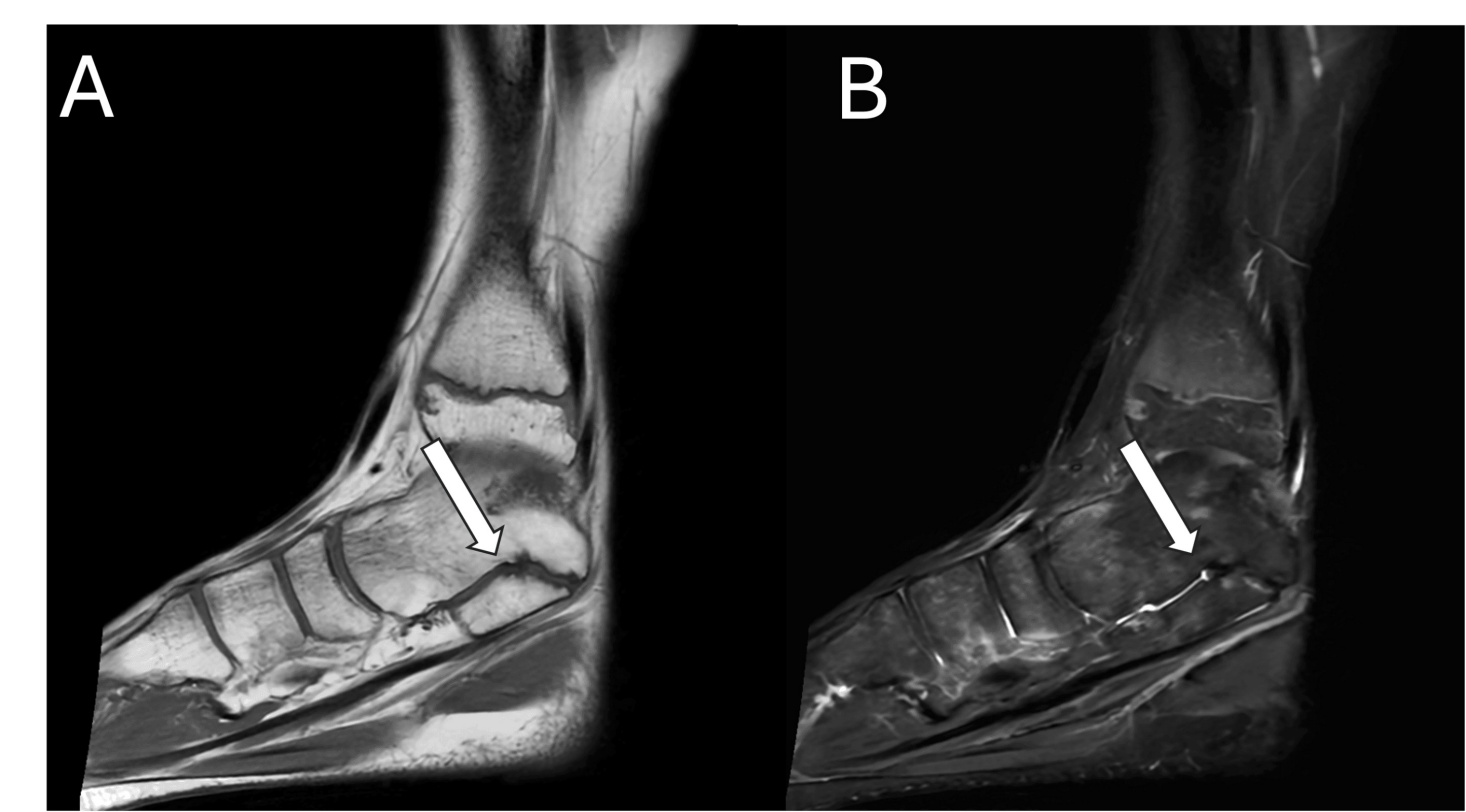
Sagittal (A) T1- and (B) T2-weighted slices of the left ankle without contrast, demonstrating a focal cortical irregularity and surrounding oedema at the medial margin of the TC joint, without continuity of the marrow signal, consistent with a non-osseous coalition shown with the white arrow.

**Figure 2:**
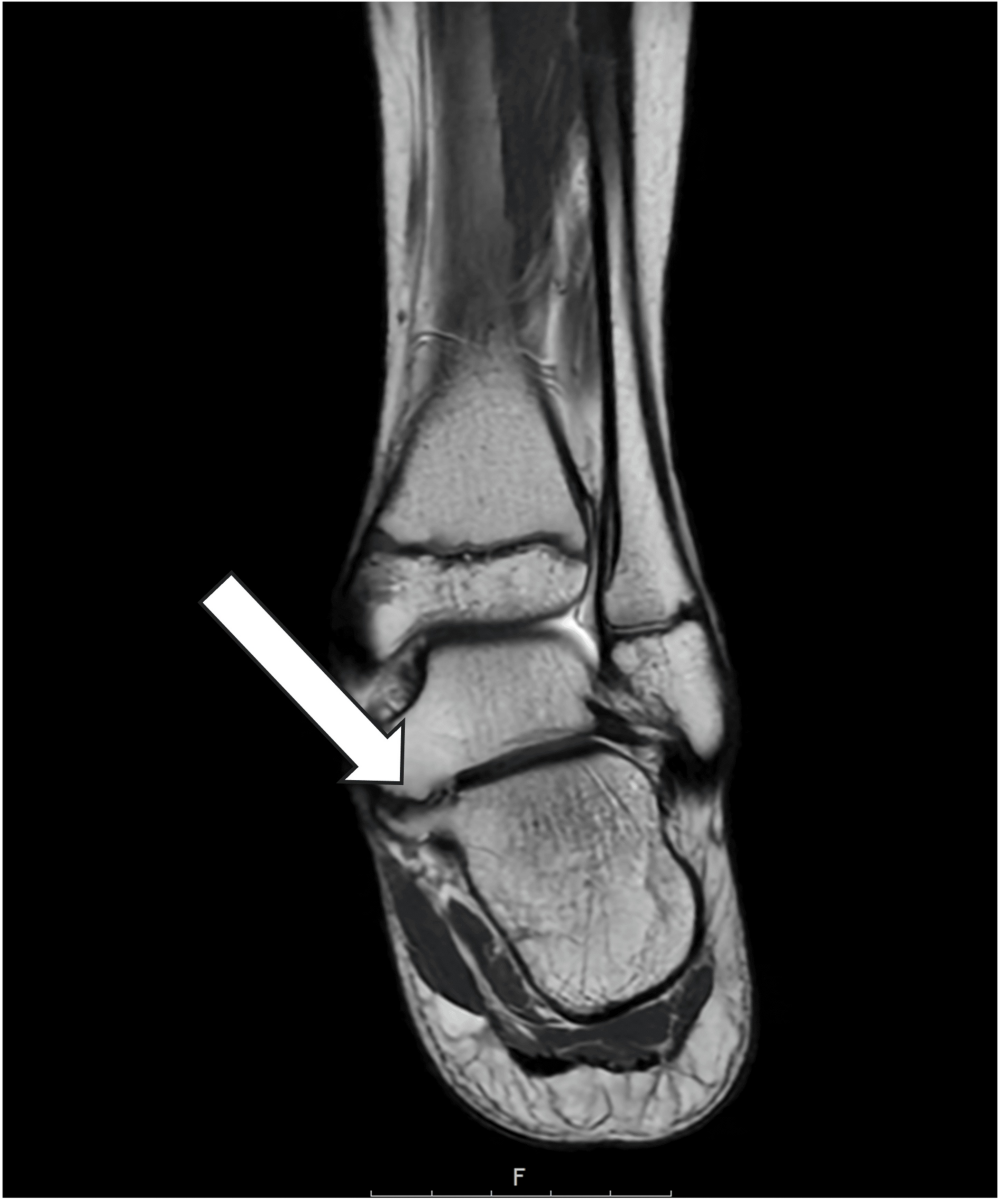
Preoperative coronal T2-weighted MRI confirming the non-osseous talocalcaneal (TC) coalition, shown with the white arrow.

Magnetic resonance imaging revealed a non-osseous, cartilaginous TC coalition, accompanied by bone marrow oedema consistent with his localised pain (Figures 1-2). Imaging confirmed preserved articular cartilage within the subtalar joint and surrounding tarsal articulations, with no evidence of secondary degenerative change or additional coalition sites.

**Figure 3:**
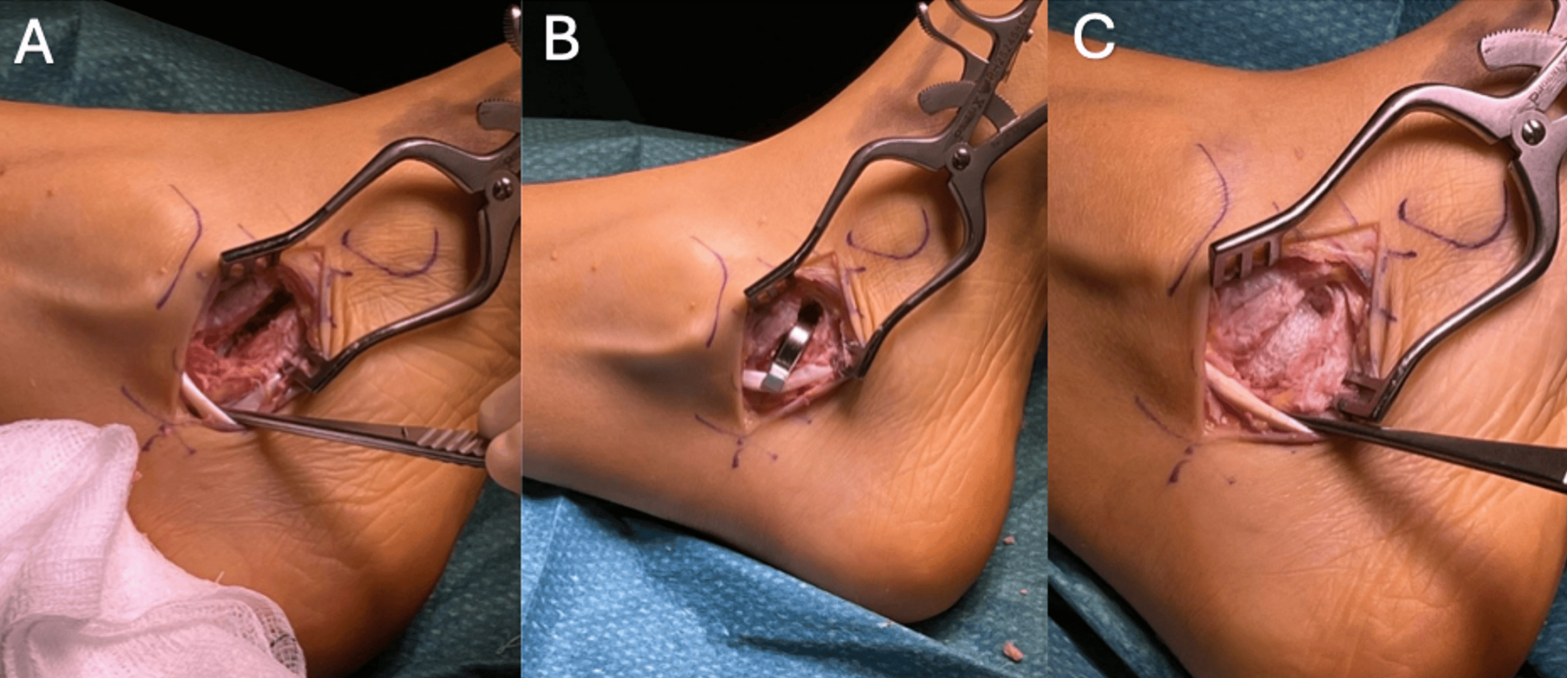
(A) Clinical intraoperative image demonstrating the medial approach to the sustentaculum tali, with excision of the pre-measured coalition block. (B) Trial sizing with RegJoint™ templates, with the 16 mm disc providing the best fit. (C) Placement of the 16 mm RegJoint™ implant within the defect, sitting securely and appearing stable. The periosteum was sutured to the edges of the spacer.

A medial approach to the TC joint was used. The tendons of tibialis posterior and flexor digitorum longus were retracted, and the coalition was completely resected by removing a 6 × 12 × 17 mm segment (Figure 3). The component was sized and trialled to ensure appropriate tension of surrounding soft tissues and recreation of hindfoot kinematics. A 16 mm RegJoint™ implant was then positioned within the defect, and its placement was confirmed intraoperatively with fluoroscopy. The wound was irrigated and closed using non-absorbable skin sutures.

Postoperative rehabilitation followed a staged protocol, beginning with three weeks of strict non-weight-bearing in a plaster cast, followed by three weeks of progressive weight-bearing in a walker boot. Supervised physiotherapy commenced at the six-week mark, with particular emphasis on restoring subtalar joint mobility and peroneal tendon strengthening. Return to competitive football was permitted at nine weeks.

**Figure 4:**
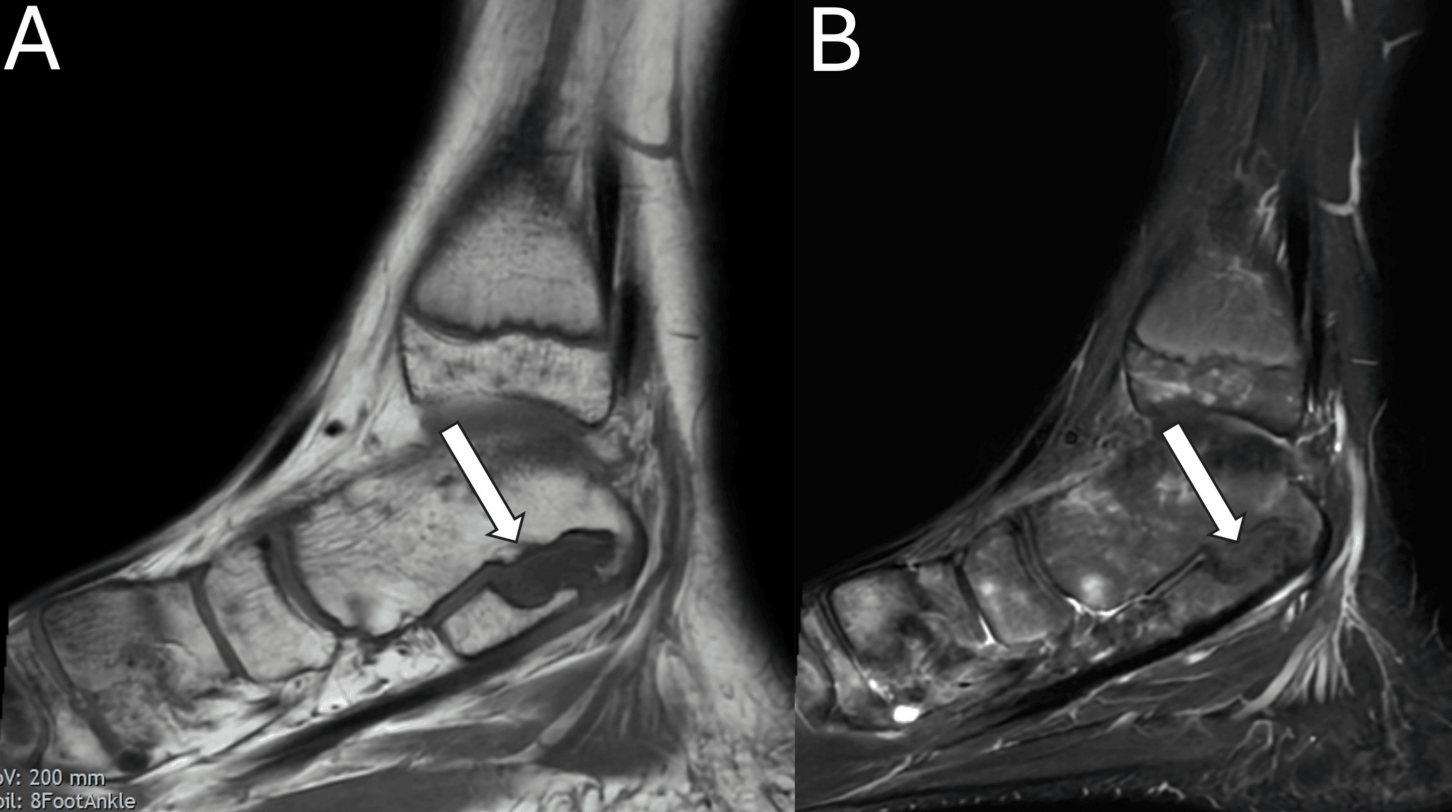
12-month post-operative sagittal (A) T1- and (B) T2-weighted slices of the left ankle without contrast showing RegJoint™ marked with a white arrow.

**Figure 5:**
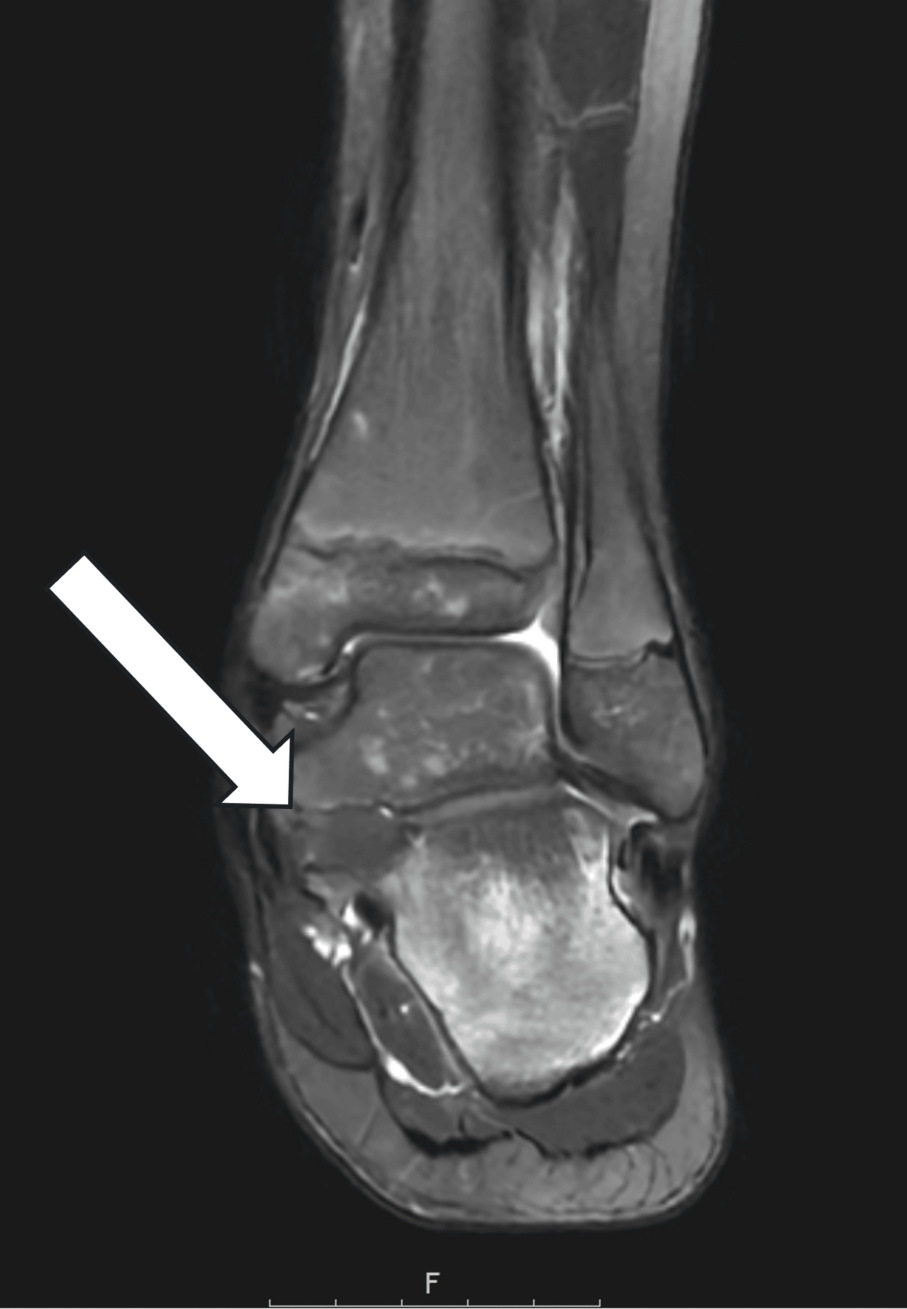
T2-weighted post-operative coronal MRI showing RegJoint™ marked with the white arrow.

Three months post surgery, the patient reported complete resolution of resting pain and full functional capacity during daily activities. He had also returned to light exercising. After six months, he was back to impact and competitive sports. An objective assessment demonstrated the restoration of the subtalar range of motion symmetrical to the unaffected contralateral side. No surgical complications or signs of coalition recurrence were evident on the MRI scans taken 12 months post surgery (Figures 4-5).

## Discussion

This novel technique in the management of TC coalition adds another management option to the surgical armamentarium. This case represents the first reported successful surgical management of TC coalition using the RegJoint™ implant, a bioresorbable porous polylactide scaffold. Previously used in small joints of the hand and great toe, this preformed device provides precise sizing, avoids donor-site morbidity, and maintains consistent joint space volume during healing. Its synthetic nature eliminates the need for autologous graft harvest, reducing surgical morbidity while maintaining a stable joint space during the healing process.

TC and CN coalitions represent about 90% of cases of all coalitions [2]. TC coalitions often present later in adolescence as ossification progresses, leading to pain, stiffness, rigid flatfoot deformity, or restricted subtalar motion.

Surgical intervention is indicated for refractory cases, with the choice of procedure guided by coalition size, joint integrity, and presence of degenerative change. For patients with preserved joint architecture and minimal deformity, resection of the coalition with or without interposition remains the preferred joint-preserving option [4].

Hubert et al. reported long-term outcomes of interposition arthroplasty using a bioresorbable PLDLA implant for end-stage hallux rigidus and arthritic hallux valgus [12]. Their study demonstrated sustained pain relief, preservation of joint motion, and high patient satisfaction, with a low complication profile and a well-tolerated implant. Radiographic follow-up confirmed maintenance of joint space and long-term functional improvement, supporting the implant’s biocompatibility and durability in small load-bearing joints. However, these findings are derived from the first metatarsophalangeal joint, which experiences different biomechanical stresses compared to the subtalar joint. The subtalar joint is subject to complex multidirectional loading and higher shear forces during gait, which may influence implant performance and longevity. Therefore, while prior evidence supports the feasibility of bioresorbable interposition arthroplasty, extrapolation to larger, more mechanically demanding joints such as the subtalar joint should be made with caution.

Various interposition materials, including autologous fat, muscle, tendon, and bone wax, have been described to reduce the risk of re-ossification [5,6]. Long-term outcomes following TC coalition resection are generally favourable. A recent systematic review reported an overall pooled success rate of 79% (95% CI: 75%-83%) for the TC coalition, with open resection achieving a success rate of 80% (95%CI: 76%-84%) and arthroscopic resection of 86% (95% CI: 71%-94%). When both arthroscopic and open resections were analysed together, procedures performed with and without interposition material demonstrated comparable outcomes, with success rates of 83% (95%CI: 78%-87%) and 79% (95%CI: 65%-88%), respectively.

Soft tissue interposition is well reported with autologous graft from the tibialis posterior tendon, demonstrating significant improvement in clinical outcomes [12].

The RegJoint™ implant offers an appealing synthetic alternative to autologous materials without the potential for donor side morbidity or the unpredictable nature of local tissues. Evidence for the use of RegJoint™ remains sparse, and despite encouraging early results, further studies are indicated to fully appraise both its efficacy and long-term outcomes. Mattila et al. published their medium-term series of trapeziometacarpal arthroplasties with RegJoint™ interposition, reporting local inflammatory reactions and osteolysis in some patients, although symptomatic relief was noted and further surgery was not indicated [13]. Similarly, in a cohort of 38 patients who underwent total trapeziectomy with RegJoint™ interposition (mean follow-up: 33 months), radiographic evidence of local bone resorption (only one case) required revision, and sustained improved clinical outcomes were recorded [14]. These findings emphasise the need for careful patient selection and long-term follow-up despite overall early positive signalling.

The technique was easy to reproduce and had a shallow learning curve, making it highly adoptable. In the lead author's experience, intraoperative fluoroscopic guidance is crucial to confirm complete resection and accurate implant placement, along with sizing. Inadequate appreciation of soft tissue tension can lead to ‘overstuffing’ of the joint, resulting in impaired kinematics. Undersizing carries the risk of recurrence or collapse of the joint, with symptoms of pain ensuing. Intraoperative assessment of loaded foot function is essential to ensure restored biomechanics.

The excellent midterm outcome observed in our patient suggests that RegJoint™ interposition may achieve at least comparable pain relief and functional recovery to traditional techniques while potentially offering technical and biological advantages. However, long-term follow-up is indicated to assess implant integration, subtalar motion preservation, and recurrence rates.

This case, therefore, represents a proof of concept demonstrating the successful application of a novel synthetic interposition technique for TC coalition. This report establishes the novel use of the RegJoint™ implant for subtalar interposition arthroplasty. Unlike traditional interposition materials such as fat graft or local tissue (e.g., tibialis posterior tendon, extensor retinaculum), which can be subject to resorption or unpredictable fibrotic scarring, the synthetic RegJoint™ scaffold offers a standardised, patient-specific geometry that may provide more consistent and durable joint spacing [6]. In this initial case, the technique facilitated a highly successful outcome, with the adolescent patient achieving complete pain resolution, restored subtalar motion, and a rapid return to exercise within three months while eliminating donor-site morbidity. To solidify this promising approach, future longitudinal and comparative multi-centre studies are now essential to evaluate its long-term durability and efficacy against these established autograft standards.

## Conclusions

This case report establishes the novel use of a synthetic RegJoint™ implant for subtalar interposition arthroplasty, demonstrating its viability as a patient-specific solution that eliminates donor-site morbidity. The technique facilitated a highly successful outcome, with the adolescent patient achieving complete pain resolution, restored subtalar motion, and a rapid return to exercise within three months.

To translate this promising pilot finding into clinical practice, future comparative, multi-centre studies are now warranted to evaluate its long-term durability against traditional autograft techniques.
